# Metabolomics improves the histopathological diagnosis of asphyxial deaths: an animal proof-of-concept model

**DOI:** 10.1038/s41598-021-89570-0

**Published:** 2021-05-12

**Authors:** Emanuela Locci, Alberto Chighine, Antonio Noto, Giulio Ferino, Alfonso Baldi, Dimitrios Varvarousis, Theodoros Xanthos, Fabio De-Giorgio, Matteo Stocchero, Ernesto d’Aloja

**Affiliations:** 1 Department of Medical Sciences and Public Health, Section of Legal Medicine, University of Cagliari, Cagliari, Italy; 2 Department of Environmental, Biological and Pharmaceutical Sciences and Technologies, University of Campania “L. Vanvitelli”, Caserta, Italy; 3 Cardiology Department, Asklepeion Hospital, Athens, Greece; 4 School of Medicine, European University Cyprus, Nicosia, Cyprus; 5 Department of Health Care Surveillance and Bioethics, Section of Legal Medicine, Catholic University of Sacred Heart, Rome, Italy; 6 Fondazione Policlinico Universitario A. Gemelli IRCCS, Rome, Italy; 7 Department of Women’s and Children’s Health, University of Padua, Padova, Italy

**Keywords:** Systems biology, Biomarkers, Medical research

## Abstract

The diagnosis of mechanical asphyxia remains one of the most difficult issues in forensic pathology. Asphyxia ultimately results in cardiac arrest (CA) and, as there are no specific markers, the differential diagnosis of primitive CA and CA secondary to asphyxiation relies on circumstantial details and on the pathologist experience, lacking objective evidence. Histological examination is currently considered the gold standard for CA post-mortem diagnosis. Here we present the comparative results of histopathology versus those previously obtained by ^1^H nuclear magnetic resonance (NMR) metabolomics in a swine model, originally designed for clinical purposes, exposed to two different CA causes, namely ventricular fibrillation and asphyxia. While heart and brain microscopical analysis could identify the damage induced by CA without providing any additional information on the CA cause, metabolomics allowed the identification of clearly different profiles between the two groups and showed major differences between asphyxiated animals with good and poor outcomes. Minute-by-minute plasma sampling allowed to associate these modifications to the pre-arrest asphyxial phase showing a clear correlation to the cellular effect of mechanical asphyxia reproduced in the experiment. The results suggest that metabolomics provides additional evidence beyond that obtained by histology and immunohistochemistry in the differential diagnosis of CA.

## Introduction

Mechanical asphyxia represents a challenging diagnosis, due to the poor sensitivity and specificity of the so-called ‘*classic signs of asphyxia*’, frequently found in non-asphyxial death autopsies. Indeed, in addition to subjective elements, such as circumstantial details and the experience of a single pathologist, the differential diagnosis of asphyxial deaths relies on histopathological analysis, extremely sensitive in identifying the signs of cardiac arrest (CA), but lacking specificity as it cannot discriminate between different causes of CA and, consequently, different manners of death. Evidence of asphyxial deaths is mainly provided through the satisfaction of an *exclusion criteria* associated with exogenous findings (*e.g.* ligature mean or strangulation marks).

A brief review of the recent literature revealed just a small number of articles addressing asphyxia through experimental protocols. Several approaches have been proposed^1–7^, mainly focused on the effects of asphyxial mechanisms, while little is known about the *real-time* biological modifications that occur during hypoxic-ischaemic insult. It was recently demonstrated^8^ that only exceptionally long asphyxial periods can produce lungs histological modifications, whilst alterations can be observed at cellular and, especially, molecular level. The consequences of mechanical asphyxia not only affect the cellular compartments but also peripheral blood homeostasis, so investigate the metabolic status of an individual exposed to this insult might improve the comprehension of asphyxial CA. A metabolomic approach appears to be a suitable tool to intercept this phenomenon.

Metabolomics studies the low-molecular-weight metabolites in a biological fluid or tissue and describes the metabolic modifications induced by any internal/external stimulus under physiological or pathological conditions. Metabolomic investigations are performed through two major analytical platforms: ^1^H nuclear magnetic resonance (NMR) spectroscopy and liquid chromatography (LC-) or gas chromatography (GC-) coupled with mass spectrometry (MS). ^1^H NMR is a versatile, fast and reproducible tool, which presents several advantages, being nonselective, nondestructive, not requiring extensive sample pretreatments and samples can be recovered for use in multiple analyses. Its main drawback is the lack of sensitivity, with a detection limit in the low micromolar range. MS needs sample separation or derivatization but is a highly sensitive method, which allows to identify and quantify a higher number of metabolites in a relatively low sample volume^9^.

A wide body of literature on the impact of hypoxic-ischaemic conditions on different biofluids, and organs, metabolome in both animal models and human studies exists, among which an important part is devoted to the study of perinatal asphyxia^10–15^ and sudden infant death syndrome^16–18^. Although scientifically remarkable, these models could not be translated to a mechanical asphyxia setup. Several molecules, such as succinate, malate, and hypoxanthine, have been proposed as potential biomarkers of the hypoxic/ischaemic phenomenon in animal models^19,20^ and human studies^21^. However, investigations on the impact of mechanical asphyxia of foremost forensic interest are still lacking.

We recently showed that metabolomics can be used to distinguish asphyxial from dysrhythmic CA in a swine model investigating plasma metabolome modifications^22^. Samples were analysed by both ^1^H NMR spectroscopy and LC–MS/MS spectrometry. Spectral data were submitted to multivariate statistical analysis. Changes in the plasma metabolomic profiles over time indicated the existence of different metabolomic patterns characteristic of the two types of CA. While the major aim of this study was to identify specific metabolomic patterns associated with ACA and VFCA for clinical purposes, the obtained results encompass latent information of foremost interest to the forensic community.

Indeed, the objective of the present work was to challenge metabolomic data with histopathological and immunohistochemical analysis of heart and brain tissues obtained after the animals’ sacrifice. Furthermore, the modifications that occur in the plasma of ACA animals during the asphyxial period were used to describe individual metabolomic trajectories so to evaluate inter-individual variability in terms of susceptibility and/or resistance to the hypoxic insult.

To the best of our knowledge, this is the first study addressing asphyxia, in which metabolomic modifications induced by a pure asphyxial mechanism were extensively investigated. Experimental evidence would be of great interest for both clinical and forensic audience.

## Results

### Clinical parameters

No statistically significant differences in haemodynamic parameters between the ACA and VFCA groups were observed during the experiment until return of spontaneous circulation (ROSC)^22^. The duration of the asphyxial pre-arrest period in ACA animals ranged from 4 to 10 min (mean value 6.8 ± 2.3 min); overall survival rate was 70% in the ACA group and 80% in the VFCA group. Among the ACA animals, 2 out of 10 did not achieve ROSC (ACA6, ACA9), and 1 out of 10 died 1 h after ROSC (ACA4). ACA5 achieved ROSC but had a final neurological score (NS) < 50 at 24 h indicating a very poor outcome. The remaining 5 animals achieved ROSC with a good outcome at 24 h (NS > 70). Among the VFCA animals, all the animals except VFCA4 and VFCA10 achieved ROSC with a good NS at 24 h. At the end of the experiment, animals that did not achieve ROSC (n = 4), died early after ROSC (n = 1), or had a final NS < 70 at 24 h (n = 1) were classified as damaged animals. The remaining animals (n = 14) were classified as no-damaged animals. Data are summarized in Table 1.

**Table 1:** Clinical and experimental parameters of the studied groups.

ACA group					VFCA group			
Animal	Mechanical asphyxia time prior to CA (minutes)	Group D vs ND	CPR time (minutes)	NS 24 h	Animal	Group D vs ND	CPR time (minutes)	NS 24 h
ACA1	4	ND	4	70	VFCA1	ND	2	100
ACA2	7	ND	2	100	VFCA2	ND	8	100
ACA3	4	ND	2	100	VFCA3	ND	4	100
ACA4	9	D	10	–	VFCA4	D	20	–
ACA5	10	D	6	50	VFCA5	ND	4	100
ACA6	8	D	14	–	VFCA6	ND	4	100
ACA7	4	ND	2	100	VFCA7	ND	2	100
ACA8	8	ND	2	100	VFCA8	ND	4	100
ACA9	9	D	9	–	VFCA9	ND	6	100
ACA10	5	ND	6	80	VFCA10	D	16	–

D = damaged animals; ND = no-damaged animals; CPR = Cardio Pulmonary Resuscitation; NS = neurological score.

### Histopathological analyses

The results of the histopathological analyses of the damaged animals were consistent with ischaemia/reperfusion injury, regardless of the study group. No major differences with regard to the amount or extent of the injuries were observed between the ACA and VFCA groups. An arbitrary scoring system was adopted, and scores were ascribed to the different specimens, depending on the main histopathological features. Oedema and hypereosinophilia were detected in all the animals, without any differences between the two groups or among animals of the same group. For this reason, these two parameters were not taken into account in the creation of the scoring system. The resulting histopathological scores are reported in Table 2.

**Table 2:** Results of histopathological and immunohistochemical analyses of brain and heart samples, with partial and global arbitrary scores.

Animal	Brain							Total Score	Heart					
	Histology				IHC					Histology			IHC	
	MI	Tu	Ty	De	S100B	GFAP	Score		Score	MI	Tu	MA	TnC	D
ACA1	0	1	0	0	1	1	3	7	4	0	1	1	1	1
ACA2	0	1	0	0	1	1	3	7	4	0	1	1	1	1
ACA3	0	0	0	0	1	1	2	5	3	0	0	1	1	1
**ACA4**	**1**	**3**	**1**	**1**	**3**	**3**	**12**	**25**	**13**	**1**	**3**	**3**	**3**	**3**
**ACA5**	**1**	**2**	**1**	**1**	**2**	**2**	**9**	**17**	**8**	**0**	**2**	**2**	**2**	**2**
**ACA6**	**1**	**2**	**1**	**1**	**3**	**3**	**11**	**23**	**12**	**1**	**2**	**3**	**3**	**3**
ACA7	0	1	0	0	1	2	4	10	6	0	1	2	1	2
ACA8	0	1	0	0	1	1	3	8	5	0	1	2	1	1
**ACA9**	**1**	**3**	**1**	**1**	**3**	**3**	**12**	**25**	**13**	**1**	**3**	**3**	**3**	**3**
**ACA10**	**1**	**2**	**1**	**1**	**2**	**3**	**10**	**19**	**9**	**0**	**2**	**2**	**2**	**3**
VFCA1	0	0	0	0	1	1	2	5	3	0	0	1	1	1
VFCA2	0	1	0	1	2	1	5	13	8	0	2	2	2	2
VFCA3	0	1	0	0	1	1	3	7	4	0	1	1	1	1
**VFCA4**	**0**	**2**	**1**	**1**	**3**	**2**	**9**	**18**	**9**	**0**	**2**	**3**	**2**	**2**
VFCA5	0	0	0	0	1	1	2	6	4	0	1	1	1	1
VFCA6	0	1	0	0	2	1	4	8	4	0	1	1	1	1
VFCA7	0	0	0	0	2	1	3	7	4	0	1	1	1	1
VFCA8	0	0	0	0	2	1	3	6	3	0	1	1	1	0
VFCA9	0	1	0	0	3	2	6	12	6	0	1	2	1	2
**VFCA10**	**0**	**2**	**1**	**1**	**3**	**2**	**9**	**18**	**9**	**0**	**2**	**3**	**2**	**2**

**IHC** = Immunohistochemistry; **MI** = Micro-Infarction; **Tu** = TUNEL; **Ty** = Tygrolisis; **De** = Demyelination; **MA** = Morphological alterations; **GFAP** = Glial Fibrillary Acid Pro; **TnC** = C-Troponin; **D** = Desmin.

Assigned scores: MI, Ty, De: **0** = absent, **1** = present; MA, **0** = absent, **1** = mild, **2** = moderate, **3** = severe; Tu: **0** = absent, **1** = up to 2%, **2** = up to 5%, **3** = more than 5%; S100B, GFAP, D, TnC: **0** = absent, **1** = mild, **2** = moderate, **3** = severe.

Heart micro-infarction was detected in 3 out of 4 damaged ACA animals, namely, ACA4, ACA5, and ACA6. The TUNEL reaction was found to be positive in 18 out of 20 animals. A TUNEL score > 1 was found in 5 ACA animals (4 damaged animals plus ACA10) and in 3 VFCA animals (2 damaged animals and VFCA2). Morphological heart alterations were more represented in the damaged animals of both groups. Notably, moderate morphological alterations were detected in 3 and 2 no damaged ACA and VFCA, respectively.

Brain micro-infarction was detected only in ACA group (namely, ACA4, ACA5, ACA6, ACA9, and ACA10). The same animals, along with VFCA4 and VFCA10, showed signs of tigrolysis. The aforementioned animals and the animal VFCA2 showed signs of demyelination. The TUNEL reaction was positive in 9 out of 10 ACA animals and 6 out of 10 VFCA animals, with higher values in damaged animals. Overall, despite damaged animals presented higher overall scores, histopathological analysis cannot distinguish between damaged animals of different groups.

### Immunohistochemical analyses

In addition to histopathological analysis, immunohistochemical staining was performed on the heart and brain tissues. Hearts were investigated for the expression of C-Troponin and Desmin, while brains were stained for GFAP and S100B. Results are summarized in Table 2.

Desmin depletion was observed in 19 out of 20 animals. Higher values appeared to be correlated with poor outcome, although some of the no-damaged animals presented a moderate (ACA7, VFCA2, and VFCA9) or severe (ACA10) loss. C-Troponin depletion was found in all the animals, ranging from moderate to severe in all the damaged animals, plus ACA10 and VFCA2, (moderate). Desmin and C-Troponin depletion was severe only in the ACA group, mild to moderate in VFCA animals.

S100B and GFAP expression was positive in all the animals, being moderate to severe in damaged ACA plus ACA10. Damaged VFCA animals and VFCA9 showed severe S100B expression, while 4 out of 10 no-damaged VFCA animals presented a moderate expression. GFAP expression was severe in 3 out of 4 damaged ACA animals and ACA10, moderate in the remaining ACA animals. Damaged VFCA animals and VFCA9 presented moderate GFAP expression while the no-damaged VFCA animals presented mild expression.

### Combined histopathological and immunohistochemical scoring

No-damaged ACA animals presented a combined score ranging from 5 to 10, while the damaged ACA animal scores ranged from 17 to 25. Notably, the no-damaged ACA10 showed combined features similar to damaged animals (total score = 19). Damaged VFCA animals had a score of 18 while no-damaged presented scores spanning from 5 to 13. The combined histopathological and immunohistochemical approach appears to be quite sensitive in the identification of tissue damage secondary to CA, as a damage threshold may be identified: no-damaged animals presented combined scores ≤ 10 (ACA) and ≤ 13 (VFCA), while damaged animals showed scores ≥ 17 (ACA) and ≥ 18 (VFCA). However, even this combined approach did not address the differential diagnosis of the two types of CA.

### Metabolomics analysis of ACA plasma samples during asphyxia

**Figure 1:**
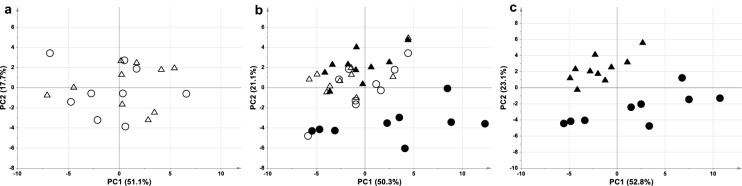
PCA score plot of ACA and VFCA plasma samples (**a**) at baseline (A = 3, Par, R^2^ = 0.81, Q^2^ = 0.59), (**b**) at baseline vs. the 5th minute of untreated CA (A = 3, Par, R^2^ = 0.83, Q^2^ = 0.68), and (**c**) at the 5th minute of untreated CA (A = 3, Par, R^2^ = 0.86, Q^2^ = 0.67). ACA samples are represented by open circles at baseline and black circles at arrest. VFCA samples are represented by open triangles at baseline and black triangles at arrest.

In our previous experiment, starting from a baseline condition of homogeneous plasma samples, two different metabolomic profiles were achieved after the induction of ACA or VFCA^22^. Here, for descriptive purposes, we show comparative PCA analysis of the samples. In particular, Fig. 1 shows the PCA score plot of ACA and VFCA samples at baseline (Fig. 1a), at baseline and at the 5th minute of untreated CA (Fig. 1b) and only at the 5th minute of CA (Fig. 1c). It can be seen that baseline samples were randomly distributed in the multivariate space, while after CA, they lay into two well separated clusters. After CA, VFCA samples did not experimented major metabolomics modifications, lying in the same space than the corresponding baseline, while ACA samples moved far away. Figure 1c shows the clear difference between the two groups of arrest samples. A pattern of metabolites responsible for the separation between ACA and VFCA samples was previously identified by the use of supervised statistical methods^22^. Specifically, higher levels of lactate, succinate, malate, fumarate, glutamate, hypoxanthine, uridine, and cytidine were detected in ACA samples, while VFCA samples maintained a metabolomic profile very similar to the samples collected at baseline. The major metabolomic modifications observed in ACA samples were clearly induced by the pre-arrest asphyxial phase, which is characterized by decreased oxygen availability and persistent blood flow.

**Figure 2:**
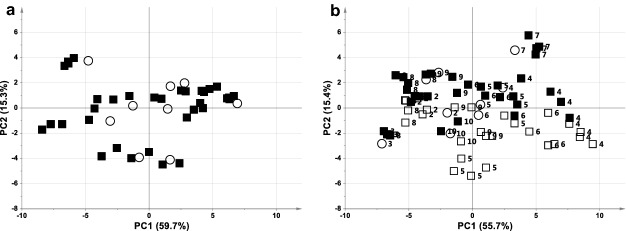
PCA score plot of ACA plasma samples collected at (**a**) baseline vs. the first 4 min of the pre-arrest phase (A = 3, Par, R^2^ = 0.84, Q^2^ = 0.73), (**b**) baseline vs. all pre-arrest samples (A = 3, Par, R^2^ = 0.82, Q^2^ = 0.71). Samples are represented by open circles at baseline, black squares at pre-arrest ≤ 4 min, and open squares at pre-arrest ≥ 5 min. Labels correspond to the animal number.

**Figure 3:**
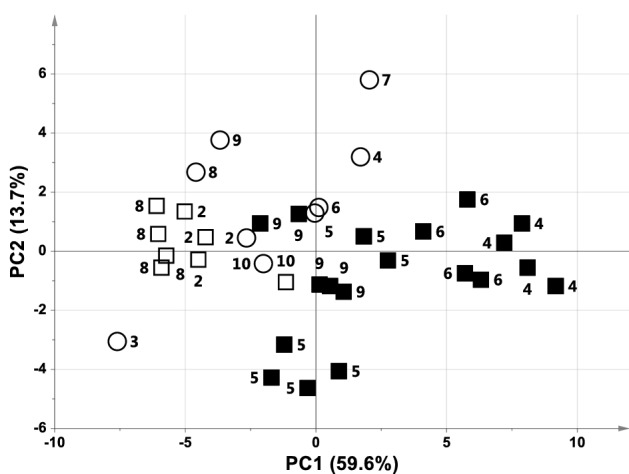
PCA score plot of ACA plasma samples collected at baseline vs. the pre-arrest samples from the 5th minute (A = 3, Par, R^2^ = 0.83, Q^2^ = 0.67). Samples are represented by open circles at baseline, black squares at pre-arrest for ND animals, and open squares at pre-arrest for D animals. Labels correspond to the animal number.

Plasma samples were collected every minute over the asphyxial period allowing to follow in detail the metabolomic behaviour of each animal during mechanical asphyxia before ACA. The PCA models of plasma samples collected at baseline and during the pre-arrest asphyxial phase are shown in Fig. 2. During the first 4 min of asphyxia plasma metabolome was not significantly modified showing a global profile similar to baseline, as samples lay in the multivariate space together with the baseline ones (Fig. 2a). Since the 5th minute of asphyxia, pre-arrest samples are gradually stratified, and several of these latter lay in the lower right quadrant of the plot, far from the other samples (Fig. 2b). These samples belong to damaged ACA animals, which showed significant metabolomic modifications during the asphyxial period. This is even more evident in a PCA model in which pre-arrest samples collected in the first 4 min of asphyxia were excluded from the analysis (Fig. 3). Interestingly, no-damaged animals with long asphyxial periods did not exhibit any significant metabolomic changes (ACA2 and ACA8).

The asphyxial phase was pivotal in identifying the asphyxiated animal with the worst prognosis. Although all the animals underwent the same insult, i.e., clamping the endotracheal tube, they reacted during the asphyxial period in different ways, with the final outcome depending on the onset of a specific metabolomic condition in this phase preceding CA. Although the limited number of experimental animals, four different groups can be defined depending on the animal’s clinical behaviour (see also Table 1):

- short asphyxial phase (4 min) and short CPR (2 min): ACA3 and ACA7 belong to this group and showed complete recovery (NS 24 h = 100).
- long asphyxial phase (7–8 min) and short CPR (2 min): ACA2 and ACA8 belong to this group (NS = 100).
- short asphyxial phase (4 and 5 min) but long CPR (4 and 6 min): ACA1 and ACA10 belong to this group and showed signs of neurological damage (NS = 70 and 80, respectively).
- long asphyxial phase (ranging from 8 to 10 min) with no or poor recovery following CPR. Damaged animals, namely ACA4, ACA5, ACA6, and ACA9 belong to this group. For the sake of precision, ACA4 was resuscitated but died 1 h after ROSC, while ACA5, which achieved ROSC after 6 min of CPR, had a NS < 50 due to severe and irreversible neurological damage.

**Figure 4:**
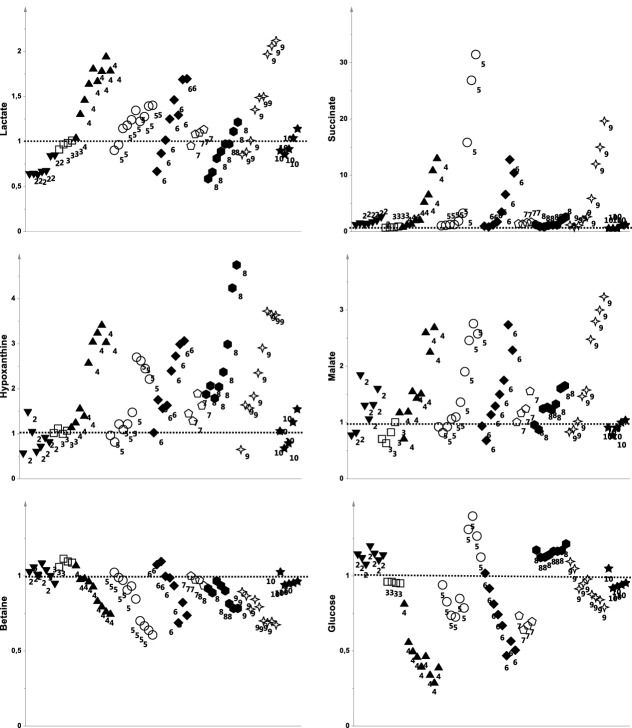
Modification of the fold change in concentration with respect to baseline values of the modified metabolites over the asphyxial period shown for each animal.

Comparison of samples collected at the end of mechanical asphyxia (last minute of the pre-arrest period) indicated that damaged ACA animals had higher levels of lactate, succinate, malate, hypoxanthine, 3-hydroxybutyrate, acetone, uridine, tyrosine, glutamate, argininosuccinate and phenylalanine and lower levels of glucose and betaine than no-damaged samples^22^. Notably, during the asphyxial period, α-ketoglutarate and glutamate, which are involved in the glutaminolysis pathway, increased in damaged animals but decreased in no-damaged ones. Although analyses of the spectra indicated a clear metabolomic profile for ACA, some metabolites appeared to drive the differentiation of damaged animals. Lactate, succinate, malate, and hypoxanthine were the most discriminant (Fig. 4).

Among the metabolites, succinate showed the highest quantitative variation. In contrast, in no-damaged animals, succinate levels remained very similar to the baseline values, reaching a maximum concentration of 1.7-fold with respect to baseline, and the overproduction of succinate was observed in damaged animals (fold change ranging from 9 to 20), suggesting the potential role of this metabolite as a biomarker. As shown, this increase in succinate is not related to asphyxial duration, while hypoxanthine showed a time-related behaviour, suggesting a possible role as agonal duration marker.

## Discussion

The main aim of this explorative experiment was to address the mechanical asphyxia conundrum from a metabolomic point of view. The differential diagnosis of asphyxial deaths can be a difficult task, as no direct asphyxial evidence is available, and the diagnosis of asphyxia may rely only on the exclusion of all other potential causes, mainly through toxicological and histological analyses. Moreover, mechanical asphyxia represents a wide and inhomogeneous group of causes of death and involves at least three major physio-pathological mechanisms with important differences in their effects on homeostasis. For these reasons, a swine animal model of mechanical asphyxia was designed, and blood samples were withdrawn every minute starting from baseline up to the 5th minute of CA and, for those that eventually survived, up to 24 h after the insult. VFCA was chosen as a control group. However, a recent experimental study demonstrated that even in ACA there is significant intra-arrest rhythm conversion and that around 70% of the animals exposed to endotracheal clamping at some point during the experiment convert to VF^23^. This experimental study may explain the heterogeneity in the metabolomic profile in some of our animals.

### Metabolomic profile at baseline

To focus on the effects of the two causes of CA, the experimental protocol was designed to limit any possible variability at baseline. Animals were extremely homogeneous, as Landrace/ Large-White pigs were of same age, weight and sex, inbreed, and were identically fed. No significant differences in clinical parameters (heart rate, mean arterial pressure, coronary perfusion pressure, cardiac output, HCO_3_^−^, pH, pO_2_, pCO_2_) were observed at the beginning of the experiment. The aforementioned reflected the animals’ metabolomes, because, as expected, no differences in the unsupervised PCA score plots of ACA and VFCA animals were detected, as plasma samples were randomly distributed in the multivariate space (Fig. 1a). This was a mandatory condition to proceed with comparison of the two CA groups. Interestingly, the PCA model for samples at both baseline and after 5 min of untreated CA revealed separation in only the ACA group samples collected at CA, while no separation was observed for VFCA samples (Fig. 1b). In other words, major modifications occurred in only the ACA metabolome (through the asphyxial and arrest periods) whereas the metabolome of VFCA animals seems to remain consistent with their baseline.

### ACA vs VFCA: histopathological and immunohistochemical features

When investigated through a routine microscopical approach, damaged animals were clearly distinguishable. A combined histopathological and immunohistochemical approach was applied to investigate heart and brain tissues.

For the immunohistochemical analysis, hearts were investigated through structural antigens Desmin and C-Troponin^24^, whose depletion in myocardial ischaemic areas has been well-described. Brains were studied focusing on S100B^25^ and GFAP^26^, markers of direct astrocyte damage. The proposed scoring system allowed us to stratify the damaged and the no-damaged animals. Notably, tissue damage was relatively equivalent between the two organs (Table 2). On the other hand, microscopic investigation did not unravel the mechanism of death, as the damaged ACA and VFCA presented comparable histopathological scores.

### ACA vs VFCA: metabolomic profile modifications

After 5 min of untreated CA, plasma metabolomes were significantly different in the two groups, as shown in Fig. 1c. Although a metabolomic profile relies on hundreds of metabolites, in our experimental results, major differences appeared to be driven by a relatively low number of metabolites. In particular, lactate, succinate, malate, fumarate, glutamate, hypoxanthine, uridine, and cytidine were significantly increased in the ACA group with respect to VFCA.

The aforementioned is of great forensic interest, as the two mechanisms of CA showed the same macroscopic and microscopic evidence. On the contrary, plasma metabolomes contained extensive information on the mechanism and, consequently, the manner of death. Notably, while tissue damage is characteristic of the ultimate step of an injury, metabolomic modifications may be identified in the earliest asphyxial phase, representing a molecular flag of the upcoming, yet still undetectable, damage. If so, the combined approach with histopathology, immunohistochemistry, and metabolomic profiling allows not only a diagnosis of the severity of the injury but also can also provide significant elements in the differential diagnosis between two of the more common causes of CA, namely asphyxia and ventricular fibrillation.

### Metabolomic profile trajectories during the asphyxial period

Differences between the ACA and VFCA metabolomic profiles at arrest were deeply influenced by the asphyxial period. This period was characterised by a considerable variation in terms of length, spanning from 4 to 10 min depending on the individual.

#### First 4 min

The metabolomic profile in this period did not show significant modifications with respect to baseline, as all the ACA animals were able to cope with the initial period of asphyxia (see Fig. 2). A possible explanation for this response may be that, despite the absence of new alveolar oxygen, blood flow is still viable, allowing peripheral oxygen extraction as in normoxia. If so, all the animals were able to survive without any metabolomic variation following the complete breathing cessation. This experimental data, if independently confirmed, may have a relevant interest in the forensic context. During cardiac arrest, blood ceases to circulate, the ensuing microcirculatory changes result in heterogeneity of perfusion and tissue oxygenation. Initially, this is partly compensated by the preserved autoregulation and the increase in the cellular metabolism rate, but at later stages, the loss of autoregulation activates the cascade of intracellular hypothermia^27^. Some of our findings could be attributed to these physiopathological hypothesis.

#### From the 5th minute: damaged animals

All damaged animals experimented a long asphyxial period, ranging from 8 to 10 min. These animals were characterised by a surge in succinate concentration (fold change = 9–20) at the end of the asphyxial period. This is consistent with what was previously described in an ischaemia–reperfusion rat model^19^. The accumulation of this TCA cycle intermediate originates from the reversal activity of succinate dehydrogenase induced by accumulated NADH which is responsible of electrons passage to the CoQ pool. This latter is the biological cause of the fumarate reduction and, finally, of succinate accumulation^28^. Notably, Complex II represents the only direct link between the TCA cycle and electron transport chain (ETC), as another indirect link is represented by Complex I. During oxygen shortage, mitochondria adjust their energetic metabolism, through TCA anaplerotic reactions, in search of alternative substrates to pyruvate. The final goal is to maintain efficient ATP production via the ETC. In the absence of oxygen, fumarate acts as the terminal electron acceptor and is rapidly reduced to succinate by the reversal activity of Complex II.

As succinate is generated, the mitochondrial dicarboxylate carriers catalyse the exchange of dianions (malate, fumarate, and succinate) across the mitochondrial membranes, balancing between mitochondrial and cytosolic succinate pools^29^. Once blood flow and oxygen supply are restored, the ETC is able to buffer the electrons generated during ischaemia, as Complex II starts to work again in a forward fashion, and Complex I, working backward, incorporates electrons carried by succinate^30,31^. Unfortunately, this mechanism can cope, in the absence of a sufficient adenine nucleotide pools, with only a limited amount of electron. Succinate overload results in a reactive oxygen species (ROS) burst and so in an irreversible cellular damage. In a rat model, 20–25 min of ischemia are needed for the reperfusion damage onset^32^.

In our model, during the asphyxial phase, animals not only experienced the ongoing hypoxia but also the persistent blood flow, and this hampers one of the protective mechanisms against ischaemia, namely the active/de-active transition of Complex I^33^, which occurs in a 10–12 min-period after the onset of ischaemia^34^. In this case, the hypoxic state is able to induce, at the same time, both the reduction of fumarate to succinate (to cope with oxygen shortage, peculiar of asphyxia) and the reverse electron flow through Complex I, determining the ROS burst (as observed during reperfusion).

Notably, the plasma metabolomic profiles of these animals presented an increase even in the malate concentration (1.6 < fold change < 2.3), suggesting an important reversal activity of the malate/aspartate shuttle. Although succinate and malate were the most significantly modified metabolites, it is worth noting that the damaged animals’ profile was also characterized by other metabolites originating from the TCA cycle anaplerotic pathways. When the two profiles were compared, the damaged animals showed an increase in α-ketoglutarate and glutamate (glutaminolysis intermediates). Such behaviour may be partially responsible for the succinate overload^35^.

#### From the 5th minute: no-damaged animals

No-damaged animals showed a variable period of asphyxia spanning from 4 to 8 min. Interestingly, ACA2 and ACA8 went through a long asphyxial period (7 and 8 min, respectively), while in the other three animals, CA occurred after a maximum of 5 min. All the no-damaged animals showed a slight rise in succinate concentration at the end of the asphyxial phase (fold change < 1.7). This finding is consistent with the more extensive role of succinate, which was recently demonstrated to act as a signalling molecule^29^. The electron buffering activity afforded by fumarate reduction to succinate serves as a pivotal mechanism of resistance to cellular oxygen shortage, as during ischaemia and hypoxia/anoxia. Below a certain succinate concentration, through the reversible activities of Complexes I and II, cells can still cope with electron production, avoiding the inevitable ROS storm once oxygenated blood flow is restored and a huge amount of succinate has accumulated, as in damaged animals.

Notably, depending on its plasma concentration, succinate seemed to act in a dual way, either modulating complex cellular functions to adapt cells to oxygen shortage (*pro-survival effect*) or boosting cellular death via an increase in ROS (*pro-death effect*). This hypothesis is supported by the well-known observation that ROS can be *good or bad* depending on their levels^30^, eventually modulated by succinate.

An interesting finding is that tissue damage/poor outcome was not directly associated with asphyxial length, as ACA2 and ACA8, despite their relatively long asphyxial period, could face asphyxia and complete recover after a short CPR period, similar to the animals belonging to the short asphyxial period (namely ACA3 and ACA7). A possible explanation for this distinctive behaviour may be represented by a metabolic rate depression, already described in molluscs^36^, and linked to vertebrates^37^. This mechanism represents a response to hypoxia based on a coordinate slowdown of both ATP production and consumption to save energy and diminish ROS and other toxic end-products accumulation. Quite recently, the ability to maintain low succinate levels during extreme hypoxic conditions was associated to hypoxia tolerance in amphibians and reptile vertebrates^38^.

Among the metabolites, hypoxanthine was the only one with a time-related behaviour, as its increase was more relevant in animals with longer asphyxial period, independently on clinical outcome and histopathological findings. As hypoxanthine is known to be a breakdown product of ATP consumption, its increase suggests that cells exhausted all of their stored energy coping with asphyxial insult, confirming previous findings of hypoxanthine as a hallmark of hypoxia^39^. Our results pinpoint a potential role of this molecule in the forensic scenario, as putative biomarker of agonal period length during mechanical asphyxia.

#### Peculiar features

Animal ACA10 was extremely interesting, as it showed a short asphyxial period without significant increase in succinate during the asphyxial and CA phases (fold change of 1.11 at the 5th minute of CA), mimicking the behaviour of no-damaged animals. This animal was the only whose metabolomic profile and histopathological and immunohistochemical features were not matching, as the latter were suggestive of an extensive tissue damage. Among the no-damaged individuals, this animal was characterized by the poorest neurological outcome (NS = 70). On the other hand, according to the metabolomic data, an important increase in succinate occurred during CPR, resulting in a fold change over 16 at ROSC^22^. These late results are quantitatively comparable to those in the damaged ACA animals, suggesting a different mode and timing of injury, probably due to a reperfusion mechanism following CPR manoeuvres.

In conclusion, modifications in plasma metabolome seem to reflect molecular mechanisms related to the asphyxial insult. The proposed ^1^H NMR approach appears suitable to be used together with histopathology and immunohistochemistry, improving their predictive ability in the differential diagnosis between asphyxial and primitive CA.

### Limitations of the study

The major limit of our study is the mandatory and shared ethical issues related to the choice of an experimental animal model. Indeed, the chosen number of animals (n = 20) was based on the guiding principles underpinning the humane use of animals in scientific research (Replacement, Reduction, Refinement—the three “Rs”). Moreover, the choice of a dysrhythmic mechanism as a control was driven by the underlying clinical purposes, as a forensic-oriented experiment would have required a healthy sham group. Our experiment was conducted on plasma samples, not allowing direct inferences regarding organ damage or behaviour from a metabolomic point of view, although succinate and hypoxanthine were the only increased metabolites in all the organs investigated in a hypoxia-reperfusion rat model^19^. Lastly, the experimental asphyxial mechanism was chosen in order to magnify the effect, at a cellular level, of progressive oxygen deprivation, early and precisely detectable with the metabolomic approach; trachea ligature cannot then intercept other pathological factors (i.e. vascular and nervous ones) contributing to the mechanical asphyxia in real-life scenario.

## Materials and methods

### Experimental protocol

The experimental protocol (approved by the Greek General Directorate of Veterinary Services; reference number 3532/04-06-2014) has been previously described^22^. The study was carried out in accordance with Utstein-style guidelines, in compliance with the ARRIVE guidelines, and all experimental protocols were approved by the Aretaieio Hospital and ELPEN Experimental-Research Center Ethics Committee. Briefly, 20 healthy female Landrace/Large-white pigs of the same age and weight were randomized into two groups, anaesthetized, stabilized for 60 min with a constant temperature of 37.5–38.5 °C in all phases, and exposed to asphyxial CA by clamping of the endotracheal tube (ACA, n = 10) or to ventricular fibrillation CA by using a pacing wire (VFCA, n = 10). In ACA animals, CA occurred after an asphyxial phase of variable duration (between 4 and 10 min). After 5 min of untreated CA, the animals were resuscitated according to the 2010 European Resuscitation Council Guidelines on Resuscitation^40^. The endpoint was ROSC or asystole. The surviving animals were monitored for 4 h while anaesthesia was maintained, without further treatment. Anaesthetics were then discontinued, and the animals were extubated. At 24 h after ROSC, a standardized neurological scoring system was used to assess the neurological status of the surviving animals. The animals were then humanely euthanized by an intravenous dose of thiopental (2 g).

Blood samples were collected from the internal jugular vein at baseline (after stabilization), at 1-min intervals until ROSC, and at 1, 2, 4 and 24 h after ROSC. Blood samples were collected in Li-heparin tubes and centrifuged at 5250*g* at 4 °C for 10 min. Plasma was separated and immediately frozen at -80 °C until instrumental analysis.

Eventually, once the animals had been humanely euthanized, necropsy was routinely performed; heart and brain samples were collected for histopathological analysis. Organ samples were stored at − 80 °C until analysis.

### Metabolomics analyses

380 plasma samples were collected during the different experimental phases, appropriately processed and analysed using ^1^H NMR and LC–MS/MS. Specifically, ^1^H NMR was used for global plasma profiling, and LC–MS/MS was used for the exact quantification of targeted metabolites. Sample preparation and analyses were performed as previously described^22^. One ACA animal (namely, ACA1) was excluded from the metabolomics analysis since the plasma samples exhibited the resonances of an exogenous contaminant.

### Statistical analysis methods

Multivariate data analysis based on projection methods was applied to NMR data as previously described^22^. Principal Component Analysis (PCA) was used to recognize patterns in the collected data while Projection to Latent Structures regression (PLS) was applied to model the time evolution of the metabolite content of the samples. Hotelling’s T2 based on the PLS after linear expansion of the timescale was used to investigate the trajectories of the plasma samples during the asphyxial period. Models were validated through sevenfold full cross-validation, as well as through permutation test on the response (1000 random permutations). Data were mean centered and Pareto scaled prior to perform data analysis. Statistical data analysis was performed by SIMCA 14 (Umetrics, Umea, Sweden), and the platform R 3.0.2 (R Foundation for Statistical Computing).

### Histopathological analyses

Specimens were fixed in 10% formaldehyde in a 0.1 M phosphate buffer solution, dehydrated, embedded in paraffin, and stained with haematoxylin–eosin, haematoxylin-van Gieson, and luxol fast blue cresyl violet. Different sections of each specimen were processed and examined with light microscopy to analyse morphology, cellular structure, nuclear integrity, and inflammatory infiltrates. Specifically, structural alterations in myocardial cells were defined as low (cellular swelling), intermediate (partial disappearance of cross-striations), or high (patchy loss or blurring of cross-striations with the absence of nuclei in most cells).

Apoptosis was evaluated through the TUNEL reaction using the peroxidase-based ApopTag kit (Merck Millipore) according to the supplier's instructions. The experiment was repeated in different sections of each specimen (two to four). At least fifty random fields at a 250× magnification per section were analysed. The apoptotic rate, expressed as the ratio of the number of TUNEL-positive cells (on nucleated cells) to the total number of cells per field, was calculated and compared in different specimens by two different observers in a double-blind fashion. Consensus on the percentage of TUNEL-positive cells was reached in all cases.

### Immunohistochemical analyses

Each specimen was cut a 5 µm thickness, places on glass and then dried overnight at room temperature. Specimens were then deparaffinized in xylene and rehydrated across a graded alcohol series, before being washed with a phosphate-buffered saline (PBS, used for subsequent washes and antibodies dilution). A 5% hydrogen peroxide was used to block endogenous activity of peroxidases. Sections were heat twice for 5 min in a microwave (700 W) in a citrate buffer (pH = 6) to perform antigen-retrieval. Antibodies dilutions 1:100 of Anti-S100 (ab14849), anti-GFAP (ab7260), anti-Desmin (ab15200) and anti-Cardiac Troponin C (ab30807) (Abcam, Cambridge, UK) were applied at 4 °C for 12 h. Sections immunostaining with the streptavidin–biotin system (Dako, Carpintera CA, USA) was then performed, using diaminobenzidine (DAB) as final chromogen and haematoxylin as nuclear counterstain. Negative controls of each tissue section were arranged excluding the primary antibody. A suitable positive control was performed with every set of slides. Experimental conditions were kept stable during the entire process. Immunohistochemical staining determination was based on intensity (0, absent; 1, weak; 2, moderate; 3, strong).

### Ethics declarations

This article does not contain any studies with human participants performed by any of the authors. The experimental protocol was approved by the Greek General Directorate of Veterinary Services (ref n. 3532/04-06-2014).

## Data Availability

All raw data are available upon request.
